# Associations between social integration, participation and productivity loss among persons with chronic pain: a registry based cross sectional study

**DOI:** 10.1186/s12891-022-05896-4

**Published:** 2022-11-05

**Authors:** Thomas Friedli, Jeannette Brodbeck, Brigitte E. Gantschnig

**Affiliations:** 1 Department of Rheumatology and Immunology, Inselspital, Bern University Hospital, and University of Bern, Freiburgstrasse, 3010 Bern, Switzerland; 2 School of Social Work, University of Applied Sciences and Arts Northwestern Switzerland, Riggenbachstrasse 16, 4600 Olten, Switzerland; 3 University of Bern, Clinical Psychology and Psychotherapy, Fabrikstrasse 8, 3012 Bern, Switzerland; 4 Zurich University of Applied Sciences ZHAW, School of Health Sciences, Institute of Occupational Therapy, Katharina-Sulzer-Platz 9, 8401 Winterthur, Switzerland

**Keywords:** Widespread chronic pain, Social context, Linear models, Work, Rehabilitation

## Abstract

**Purpose:**

To examine associations between factors of social inclusion and participation and productivity loss in employed persons with chronic pain, assessed for an interprofessional pain rehabilitation programme. We hypothesized that factors of social inclusion and participation and work related social factors are significantly associated with productivity when experiencing chronic pain and we expected a moderate effect.

**Methods:**

Cross-sectional study using data collected prospectively in an interprofessional patient registry for chronic pain. The primary end point was productivity loss, measured with the iMTA Productivity Costs Questionnaire. We included data from 161 individuals. To be included, persons had to be 18 years old or older, in paid work, and had to have a medical diagnosis of chronic pain syndrome with actual or potential tissue damage. In addition, participants had to have indicators of significant impairments in psychosocial functions.

**Results:**

Linear regression analysis showed that a highly stressful professional situation, frequent problems regarding the compatibility of the family and job and not being Swiss were associated with a significantly higher total productivity loss. Similar results were found for productivity loss in paid work. However, problems concerning the compatibility of the family and job did not reach the significance level for productivity loss in paid work.

**Conclusion:**

The results of this study underscore the importance of factors of social inclusion and participation for interprofessional rehabilitation programmes to manage chronic pain especially when focussing on productivity loss.

## Introduction

Chronic pain (CP) is a major challenge worldwide, not only for those directly affected, their families and their professional environment [1], but also for the health care system, the economy and public health [2–5]. On an individual level, chronic pain affects physical and mental functioning [6–8], reduces quality of life [2] and productivity [9, 10]. In Europe and the USA, about one in five persons suffer from chronic pain [11, 12]. This corresponds to around 95 million people in the EU and around 50 million people in the USA. CP is thus one of the main causes of inability to work and – on a societal level – is responsible for enormous direct medical costs and loss of productivity [11, 12]. In our understanding, productivity or the loss of it due to illness measured in hours is the measurable outcome of work ability. Productivity loss in paid work includes both absenteeism and presenteeism. However, it should be noted that many closely related but different concepts are used in literature to measure the work-related outcome of rehabilitation programmes (e.g., ability to work, return to work, productivity, employment success). This fact complicates the comparability and interpretation of results.

One of the main groups of CP is chronic musculoskeletal pain (CMP) [13]. As a complex biopsychosocial phenomenon, CMP is highly demanding both for interprofessional interventions [1, 14, 15] and in research. In chronic pain research, social factors have received little attention. The authors of recent studies largely agree that social factors play an important but previously underestimated role in both the aetiology of CMP and concerning outcomes of rehabilitation programmes such as productivity despite CMP [1, 14, 16–18]. We define social factors as factors that characterize individuals as an integral part of their environment at different societal levels. At the societal micro and meso level, this means inclusion in personal networks as well as a fulfilling professional situation.

In this study, particular focus was placed on the association between productivity and social factors related to the individuals work situation as well as social inclusion and participation. Our previous research has shown that inclusion in the family and a circle of friends and the social situation at the workplace are experienced by those affected as significant factors influencing the sustainability of the outcomes of our interprofessional outpatient rehabilitation programme [19].

In recent studies, the following social, socioeconomic or sociodemographic factors have been proven to influence productivity / the ability to work / the return to work of persons with CMP: disability or social security pension status [14, 17, 20], significant others/ family [21, 22], educational status [21, 23, 24], the workplace situation and related job perceptions [25], residence status [26], socioeconomic status [23, 24] and social functioning in general [16]. However, most of the recent studies focus on the return to (paid) work and few on productivity or productivity loss. Thus, little is known about the association between social factors such as social inclusion and participation and work-related social factors and the productivity of persons with chronic pain.

The knowledge about the role of social factors is of central importance for the development and advancement of specialized interprofessional pain rehabilitation programmes. Thus the research question of this study was as follows: Which social factors are associated with productivity loss among individuals who are in paid work and beeing assessed for an interprofessional pain rehabilitation programme?

Based on the state of research and our experience in rehabilitation practice, we hypothesized that the factors of social inclusion and participation and work-related social factors are significantly associated with productivity in chronic pain and expected a moderate effect.

## Methods

### Study design and setting

This is a cross-sectional study using data collected prospectively in a patient registry for chronic pain. The study was conducted at the Department of Rheumatology and Immunology of the Inselspital, Bern University Hospital in 2020. The services offered by the Department include interprofessional outpatient and inpatient rehabilitation programmes for patients with CMP. In this study, we used baseline-data of patients evaluated for participation in these rehabilitation programmes. Criteria for inclusion were (1) age between 18 and 75 years; (2) diagnosis of chronic musculoskeletal pain syndrome according to ICD-10 [27] with chronic pain either (a) associated with actual or potential tissue damage or (b) associated with tissue damage and a mental disorder; (3) indicators of significant impairment in psychosocial functions. The exclusion criteria were: (1) a primary mental disorder, (2) refusal to participate in an interprofessional outpatient rehabilitation, (3) and limited skills in German language.

### Database and data collection

For this study, we used health-related data from a patient registry for chronic pain (Chronic Pain Registry). The Chronic Pain Registry was developed in 2017/2018 as an internal clinical registry and was implemented in May 2018 following international guidelines and national recommendations for the development and operation of health-related registries [28]. It was the first interprofessional registry for persons suffering from chronic pain in Switzerland [29]. One of its aims is to analyse the associations of risk factors and impairments. For the Chronic Pain Registry, we used REDCap [30] with an integrated audit trail as an electronic data capture software and database platform. For entering survey data, participants were given a tablet computer in which to enter their survey data. A personalized QR code gave them access to the corresponding assessments in REDCap.. To ensure the highest possible quality of measurements, we developed a step-by-step guide in simple language and scheduled sufficient time in the treatment plan to complete the assessments. For entering the results of clinical tests, we trained designated members of the interprofessional team and gave them direct access to REDCap.

### Measures

#### Productivity loss

The primary endpoint for this study was the iMTA Productivity Cost Questionnaire (iPCQ) [31] to evaluate the productivity loss of persons with chronic pain. Participants self-rated their productivity loss using the iPCQ. For this purpose, productivity loss due to absenteeism, presenteeism, and unpaid work were calculated using a given formula and added together to obtain a total score of productivity loss. The iPCQ is an internationally standardized, generic (diagnosis-unspecific) assessment tool for recording disease-related productivity loss, including absenteeism, presenteeism, and unpaid work. Although the iPCQ is a relatively new instrument, it has already been internationally validated [32–34]. To ensure comparability, we only used data on short-term productivity loss (productivity loss in the last 4 weeks) in the calculations.

#### Social factors

Due to a lack of a standardized and validated questionnaire for all social factors, we created a tailored Case Report Form (CRF), in which the relevant factors were assessed using items from three large national and international surveys to ensure the quality and validity of single questions. These surveys are the Swiss Household Panel [35], the Swiss Health Survey [36], and the Survey of Health, Ageing and Retirement in Europe [37]. The items assessed are the perception of occupational situation, the compatibility of family and job, and inclusion in personal networks as indicators for the inclusion in communities and residence status as an indicator for societal participation.

#### Pain

Participants rated their average pain intensity over the previous 7 days using a digital Visual Analogue Scale (VAS) from 0 (no pain) to 100 (worst pain). Both paper-based and digital VAS are well-validated measures in self-assessing pain intensity [38]. The digital VAS in REDCap measures subjective pain by asking the participants to mark their perception of pain on a line with 100 units. They have to mark the point by moving a digital slider. The selected slider position correspondents to a score ranging from 0 to 100, where higher scores indicate greater intensity of pain.

#### Demographics

Participants responded to questions about net income per person in their household, and educational level. Information about sex and age were extracted directly from the clinic information system into the registry. For educational level, we recoded the values in three groups (ISCED 0 and 1, ISCED 2–4, ISCED 6–8) and treated them as dummy variables in the regression analysis because of the educational situation in Switzerland and the nominal scale of the variable.

### Ethics approval and trial registration

The use of anonymized data for this study was approved by the Ethics Committee of the Canton of Bern in December 2018 (Project-ID 2018–01583). For this study we strictly followed the principles of biomedical [39] and social science ethics [40]. All patients were informed verbally and in writing about the objectives of a patient and quality registry and the benefits and risks of the study. All participants signed an informed consent form prior to inclusion. Data collection, transmission, storage, and analysis within this study were conducted strictly according to the legal requirements of the Swiss government [41, 42].

### Data diagnostics and preparation

**Fig. 1:**
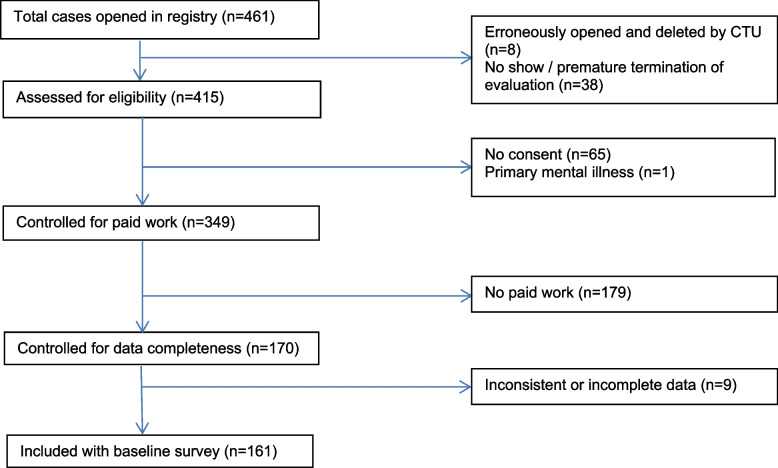
Flowchart of included cases

From a total of 461 sets of health-related person data opened in the registry, 349 were eligible for this study, and 170 of these indicated that they were in paid work. In 49 cases, we conducted follow-up telephone interviews to complete or check data for consistency. Most, but not all of the inconsistent or incomplete data sets could be completed or validated as a result. This resulted in 161 participants in this study. For details, please see Fig. 1.

Before and after conducting the regression analyses, we tested data for violations of assumptions (pre-analysis-testing of data and post-analysis-testing of residuals): Pre-testing showed no non-linear correlations in scatterplots and no strong (*r* < .8) correlations between scaled variables (no multicollinearity). We winsorized two extreme cases in the variable “net income per person in household”. The testing of residuals after regression analysis showed independence of residuals with Durbin-Watson-statistics of 2.04 for total productivity loss, and 2.00 for paid work productivity loss. Tests for outliers showed that the values of the residuals were within the limits. The highest VIF was 4.05 for both models indicating no multicollinearity, but P-P-diagram and scatterplots showed violations of the assumption of normal distribution of residuals and homoscedasticity for both models. Based on these results, the regression analyses were performed again using bootstrapping.

### Statistical analyses

We conducted all statistical analyses using SPSS v.25 with fix-packs 1–3. We calculated descriptive statistics for productivity loss, age, sex, educational level, net income per person in household, pain intensity, stress in professional situation, compatibility family-job, satisfaction with personal relationships, and residence status. For parametric correlations we performed Pearson correlation index and for nonparametric correlations Spearman’s-Rho in order to examine the correlation between productivity loss and social and demographical factors.

Since we wanted to know what additional variance was explained by the social factors beyond that explained by demographic variables and pain, we conducted two hierarchical multiple regression analyses to examine in detail the relationship between selected variables of social inclusion with participation in chronic pain conditions. In order to provide methodologically sound statements, we calculated the models for total productivity loss separately from productivity loss at paid work.

The relationship between productivity or work ability, chronic pain [9, 10] and socioeconomic factors [43, 44] is well known from the literature. Since we suspected that social factors explained additional variance, we included the demographic factors (age, sex, net income per person in household, and educational level) into a first block, VAS pain into a second block, and the social factors into a third block.

The third block of predictor variables consisted of three subjectively rated social variables (stress in professional situation, satisfaction with social relationships, compatibility of family and job), and residence status. Beta values with 95% confidence interval, standardized b and *p* values for all variables were calculated. For each step in the model, explained variance (R^2^ and R^2^-change) were calculated.

## Results

### Participant demographics

Participants included 161 adults with CMP who were assessed at the University Hospital Bern, Department of Rheumatology and Immunology between May 2018 and July 2020 and who would potentially participate in one of the clinic’s interprofessional rehabilitation programmes. The participants were predominantly female (71.4%, *n* = 115). The average age was 44.68 years (*SD* = 11.88, range 18–63). Most participants had a lower (20.5%; *n* = 33) or upper secondary education qualification (including an apprenticeship qualification) (47.8%, *n* = 77) and 22.3% (*n* = 36) had a tertiary degree (bachelor’s or higher). The mean net income per person in the household was about 2185 USD (*SD* = 1944 USD). compared with the median net income in Switzerland of 4559 USD per month in 2019 [45]. Mean pain intensity for participants in the week before admission to hospital was 65.2 (*SD* = 17.5) on a digital VAS-scale from 0 (no pain at all) to 100 (worst pain). The average workload of the participants in the 4 weeks before hospitalization was 32.43 hours per week (*SD* = 12.14). Complete details on participant demographics are presented in Table 1.

**Table 1:** Sociodemographic characteristics of participants

Characteristic	*n*	%	*M*	*SD*
Sex				
Male	46	28.6		
Female	115	71.4		
Residence status (n, %)				
Swiss nationality	139	86.3		
Permanent residence permit	13	8.1		
Limited residence permit	8	5		
unknown	1	0.6		
Highest educational level (n, %)				
Uncompleted compulsory education	8	4.9		
Lower secondary education qualification (completed compulsory education)	33	20.5		
Post-secondary, non-tertiary education qualification	7	4.3		
Higher secondary education qualification, including apprenticeship	77	47.8		
Teriary degree (Bachelor’s or higher)	36	22.3		
Age			44.6	11.8
Net income per person in household in US dollars			2963	2008
Average pain intensity on VAS Pain (last week)			65.2	17.5
Average work load in hours per week			32.43	12.14

*N* = 161

### Associations between social factors and productivity loss

Bivariate correlation analyses revealed significant associations between total productivity loss and stress in professional situation (*r*_*s*_ = .593; *p* = < .001), difficulties with the compatibility family-job (*r*_*s*_ = .192*, p* = .003), residence status (*r* = −.197; *p* = .0012), and sex (*r* = .212, *p* = .007).

No significant correlations were found between age, net income per person in household, pain intensity, satisfaction with familial relationships, satisfaction with relationships in social environment, participation in social groups and educational level.

Productivity loss in paid work correlated significantly with stress in professional situation (*r*_*s*_ *=* .471*, p* = < .001), residence status (*r* = − 208, *p* = .008), and sex (*r* = .294, *p* = < .001). In contrast to the total productivity loss, the compatibility family-job was not significantly associated with productivity loss in paid work. No significant correlations were found for age, net income per person in household,pain intensity, satisfaction with familial relationships, satisfaction with relationships in social environment, participation in social groups, and educational level. For all details on bivariate correlations, please see Table 2.

**Table 2:** Correlations for study variables

Variable	1	2	3	4	5	6	7	8	9	10	11
1. Total productivity loss											
2. Productivity loss in paid work	.935**										
3. Sex	.212**	.294**									
4. Age	.151	.117	−.060								
5. Net income per person in household	−.099	−.088	.246**	.049							
6. Educational level (index)	−.058	−.138	−.025	.004	.272**						
7. Pain intensity (last week)	−.062	−.050	−.077	.130	.074	−.134					
8. Stress in professional situation	.539**	.471**	.101	.114	−.162	−.081	.063				
9. Compatibility of family and job	.192*	.112	−.110	.001	−.175	−.077	.089	.457**			
10. Satisfaction with relationships in social environment	−.083	−.133	−.026	.036	.017	−.052	−.086	−.014	−.126		
11. Participation in social groups	−.156	−.110	−.048	.004	−.074	.076	.088	−.190*	.002	.180*	
12. Residence status	−.197*	−.208**	.037	−.082	−.010	.138	−.023	−.098	.045	−.047	.073

*p < .05. **p < .01

A series of linear regression analyses were then performed examining the significant correlations of the above-mentioned variables with productivity loss at paid work and total productivity loss. In the first step, sex, age, and uncompleted compulsory education correlated significantly with productivity loss (see Tables 3 and 4). Whereby male sex, higher age and uncompleted compulsory education predicted higher productivity loss in patients with chronic pain (see Tables 3 and 4). Including the intensity of pain in the models as a second step did not significantly increase the amount of variance explained and pain was not significantly associated with productivity loss, either in paid work or in total (*ΔR*^*2*^ = .000 for productivity loss at paid work, .001 respectively for total productivity loss). However, including the variables of social inclusion and participation in the models resulted in a significant increase of explained variance (see Tables 3 and 4). A highly stressful professional situation, frequent problems with the compatibility of the family and job and not being a Swiss national predicted a significantly higher total productivity loss. Similar results were found for productivity loss in paid work. However, problems concerning the compatibility of the family and job did not reached the significance level for productivity loss at paid work.

**Table 3:** Hierarchical regression results for total productivity loss, bootstrapped (*N* = 161)

Variable	*B*	95% CI for *B*		*SE B*	β	*Sig. (2-tailed)*	*adj. R*^*2*^	*ΔR2*
		*LL*	*UL*					
Step 1								
Constant	36.65	−7.35	89.91	24.91		.149	.078	.111
Age	1.43	0.29	2.37	0.52	0.21	.008		
Sex	39.86	9.85	71.44	15.39	0.23	.014		
Net income	−0.01	− 0.01	0.00	0.00	−0.15	.092		
EduLev1	33.82	−26.61	91.14	30.32	0.09	.242		
EduLev2	0.18	−29.70	34.19	16.17	0.00	.987		
Step 2								
Constant	46.38	−17.88	121.71	35.63		.195	.073	.001
Age	1.45	0.35	2.36	0.52	0.21	.006		
Sex	39.21	8.93	71.21	15.49	0.22	.016		
Net income	−0.01	−0.01	0.00	0.00	− 0.15	.111		
EduLev1	34.98	−30.04	91.16	30.38	0.09	.218		
EduLev2	−0.89	−30.95	31.43	16.41	0.00	.960		
Pain intensity	−0.17	−0.91	0.60	0.38	−0.04	.671		
Step 3								
Constant	111.03	25.85	217.30	49.91		.021	.309	.275
Age	0.52	−0.61	1.46	0.52	0.08	.339		
Sex	29.53	4.17	57.57	13.54	0.17	.034		
Net income	0.00	−0.01	0.00	0.00	− 0.09	.251		
EduLev1	4.85	−60.53	68.80	34.05	0.01	.896		
EduLev2	−2.09	−32.55	27.55	15.31	−0.01	.891		
Pain intensity	−0.35	−1.04	0.26	0.31	−0.08	.263		
ProfSit = rather fulfilling	13.48	−54.49	82.32	32.94	0.05	.678		
ProfSit = partly-partly	16.00	−31.11	60.87	23.39	0.08	.483		
ProfSit = somewhat stressful	1.93	−46.30	48.36	23.63	0.01	.932		
ProfSit = strongly stressful	89.54	43.40	134.26	22.55	0.56	.001		
CompFamJob = rarely	−23.46	−83.10	42.13	31.43	−0.08	.455		
CompFamJob = sometimes	−11.99	−58.21	30.80	22.88	−0.05	.598		
CompFamJob = mostly	−41.92	−82.24	−4.89	20.02	−0.23	.037		
CompFamJob = always	−18.20	−55.29	19.78	19.58	−0.11	.363		
SatSoc	−0.19	−0.76	0.41	0.29	−0.06	.520		
Residence status	−36.29	−75.34	−3.98	18.12	−0.16	.045		

*CI* Confidence interval, *LL* Lower limit, *UL* Upper limit, *Net income* Net income per person in household, *EduLev1* Educational Level 1 (uncompleted compulsory education), *EduLev2* Educational Level 2 (secondary education), *ProfSit* Professional situation, *CompFamJob* Compatibility family and job, *SatSoc* Satisfaction with social relationships

**Table 4:** Hierarchical regression results for productivity loss at paid work, bootstrapped (*N* = 161)

Variable	*B*	95% CI for *B*		*SE B*	β	*Sign. (2-tailed)*	*adj. R*^*2*^	*ΔR2*
		*LL*	*UL*					
Step 1								
Constant	30.35	−9.58	78.38	22.39		0.174	.113	.144
Age	1.04	0.00	1.87	0.48	0.17	0.032		
Sex	46.01	20.60	72.47	12.90	0.29	0.002		
Net income	−0.01	− 0.01	0.00	0.00	−0.15	0.076		
EduLev1	48.92	−7.79	92.00	25.40	0.15	0.040		
EduLev2	−1.93	−32.08	29.05	15.21	−0.01	0.907		
Step 2								
Constant	33.94	−22.27	103.89	32.23		0.297	.107	.000
Age	1.04	0.01	1.87	0.48	0.17	0.030		
Sex	45.77	20.48	72.34	13.16	0.29	0.002		
Net income	−0.01	−0.01	0.00	0.00	− 0.15	0.078		
EduLev1	49.35	−4.13	91.66	25.56	0.15	0.039		
EduLev2	−2.32	−32.18	29.69	15.45	−0.01	0.888		
Pain intensity	−0.06	− 0.77	0.56	0.33	−0.02	0.844		
Step 3								
Constant	105.10	23.88	215.58	46.60		0.030	.388	.244
Age	0.26	−0.81	1.18	0.50	.04	0.606		
Sex	38.12	15.59	61.67	11.70	.24	0.001		
Net income	0.00	−0.01	0.00	0.00	−.10	0.193		
EduLev1	24.52	−33.22	81.22	30.20	.07	0.400		
EduLev2	−2.83	−31.65	25.24	14.21	−.02	0.844		
Pain Intensity	−0.23	−0.85	0.32	0.29	−.06	0.412		
ProfSit = rather fulfilling	3.09	−48.72	54.13	26.62	.01	0.900		
ProfSit = partly-partly	1.45	−44.58	43.26	22.19	.01	0.943		
ProfSit = somewhat stressful	−12.50	−59.02	26.50	22.06	−.05	0.544		
ProfSit = strongly stressful	63.36	19.47	104.70	21.66	.45	0.002		
CompFamJob = rarely	−11.99	−72.75	47.28	29.88	−.04	0.670		
CompFamJob = sometimes	−8.91	−46.23	30.35	19.66	−.04	0.628		
CompFamJob = mostly	−33.56	−68.51	−0.35	17.60	−.21	0.057		
CompFamJob = always	−12.75	−47.17	24.11	18.04	−.09	0.462		
SatSoc	−0.22	−0.67	0.23	0.24	−.08	0.378		
Residence status	−33.16	−62.85	−6.36	14.33	−.16	0.022		

*CI* Confidence interval, *LL* Lower limit, *UL* Upper limit, *Net Inc.* Net income per person in household, *EduLev1* Educational Level 1 (uncompleted compulsory education), *EduLev2* Educational Level 2 (secondary education); *ProfSit* Professional situation, *CompFamJob* Compatibility family and job; *SatSoc* Satisfaction with social relationships

## Discussion

The primary aim of this study was to evaluate the association between productivity loss and factors of social inclusion and participation among adultss with CMP who were in paid work. Our results showed that the three factors stress in professional situation, compatibility of the family and job, and residence status explained a significant part of total productivity loss and of productivity loss in paid work. However, pain intensity was not associated with productivity loss.

Our findings support the results of earlier studies that factors of social inclusion and participation had an effect on productivity or the ability to work of persons with chronic pain [1, 16, 21].

In contrast to some other studies [46, 47] but in line with de Vries et al., and Lillefjell [48, 49], pain was not significantly associated with productivity loss. The divergent study results regarding pain could be explained by the fact that personal or work-related variables (in the case of de Vries et al. and Lillefjell et al.) or variables of social inclusion and participation may have moderated the effect of pain and that the studies with divergent results include a general working population and not exclusively persons with chronic pain.

In our data, we found a moderate association between the experience of a stressful professional situation and productivity loss. In this context, it seems important that experiencing a professional situation as stressful is more than a result of an individual’s lack of coping strategies, as it is also a result of structural / institutional workplace conditions. Earlier studies found that structural (system-related) job factors affected the experience of professional situations as stressful or burdensome [50, 51]. These include factors of social inclusion and participation like the employers attitudes, lack of co-worker support, lack of institutional resources for workplace adaptation, or social prejudice and stereotypes [51, 52]. Jetha et al. (2019) showed that a lack of institutional workplace support has a significant negative impact on the productivity of people suffering from a rheumatic disease [53]. With our study, there is growing evidence that stressful professional situations such as a lack of support from the employer or lack of understanding from work colleagues may negatively affect the productivity of people with chronic illnesses. Therefore, the requirements and knowledge of employers should be taken into account in the process of setting interprofessional rehabilitation goals, of planning and implementing interventions.

To our knowledge, there are no studies to date that have examined associations between the compatibility of family and job and productivity or ability to work in the case of chronic pain.

In our sample, the compatibility of family and job was significant only for total productivity loss, but not for productivity loss in paid work. We interpret this fact in the sense that people with chronic pain tend to invest all their remaining energy in paid work and, therefore, lose productivity in unpaid work. This interpretation is supported by the results of a qualitative study [19]. The significant association evident in our data may be related to Switzerland’s relatively poorly developed family policy [54]. The supply of childcare places is insufficient to meet demand [55]. In addition, parental contributions for a childcare place are very high, which makes access particularly difficult for lower-income families [54].

According to the Swiss Federal Statistical Office (BFS), in contrast to most other European countries, most employed persons with care responsibilities cite at least one important obstacle to reconciling family and work. In most EU/EFTA countries, however, a clear majority does not see any obstacles in the reconciliation of family and work [56]. Against this background, the results of the study, in which more than half of the participants live with children, are not surprising. Considering that reconciling family and work is demanding in itself, it can quickly become overwhelming in the case of chronic illness and a poorly developed state childcare system, leading to productivity losses, both for paid and unpaid work. It would be interesting to see whether the compatibility of job and family has less impact on productivity in chronic pain in countries with a better developed public childcare system.

In our data, residence status also correlates with productivity loss. Specifically, individuals without Swiss citizenship have higher productivity losses from chronic pain than Swiss citizens. This is in line with the findings of Hamer et al. [26] on workers with chronic pain in Canada. Like Hamer et al., we assume that this association is related to the type of work (jobs that are easily accessible to people who are not Swiss nationals are often physically very demanding),to barriers in the healthcare system (language barriers, lack of information services) and social barriers (lack of supportive networks). However, it is essential that this relationship be examined in more detail from a social science perspective in future studies in order to understand its complexity. Only a precise understanding of this association allows an appropriate adaptation of the interventions in interprofessional programmes.

In summary, in our sample of patients with chronic pain, we found associations between productivity loss and residence status, stress in the professional situation, and the compatibility of family and job. These factors of social inclusion and participation explain a significant part of the productivity losses in persons with CMP and, therefore, need to be addressed in interprofessional pain rehabilitation programmes to ensure the long-term benefit of such interventions. In practice, this can be implemented, for example, through a careful and comprehensive social diagnosis, help in finding a job adapted to the persons’s health situation, support for supplementary childcare or social work family counselling. In order to effectively, efficiently and sustainably address these systemic and work-related factors, interprofessional pain rehabilitation teams should, therefore, include specialists for systemic interventions such as clinical social workers and occupational therapists.

Our results are primarily valid for persons of working age in paid work who suffer from chronic pain and have been assessed for rehabilitation in our clinic. Since our clinic is a central hospital with a large geographical catchment area (e.g. urban and rural regions), it can be assumed that our sample represents well the total population of people with chronic pain in paid work in Switzerland.

The major strength of this study is that it is one of the first to examine the relationship between productivity and factors of social inclusion and participation. It thus provides indications that relatives and employers must be involved in the professional rehabilitation process, and that the factors mentioned should be in focus in interprofessional rehabilitation programmes. Involving employers means considering their knowledge and requirements, e.g. by providing information about support options through social insurances. This should enable them to support people with chronic pain at the workplace, but also meet the requirements of the company.

### Limitations

Nevertheless, several limitations should be considered when interpreting the findings from this study. First, with our cross-sectional data, it is not possible to determine causality or the direction of the relationships. For example, lack of participation can affect productivity, but low productivity at work and the resulting feelings of stress and demoralisation can conversely undermine personal relationships and lead to strain on work and family. Prospective studies are needed to explore these associations in more detail. A second limitation is the use of the German version of the iPCQ as an outcome measure. The German version of the iPCQ has been translated by the developers, but has not yet been culturally validated for use in Switzerland. Some terms in the questionnaire are not adapted to country-specific terminology and could lead to difficulties in completing the questionnaire (e.g. the German word “schaffen” for “to achieve something” means “to work” in Swiss German).

The high dropout rate is a further limitation. However, this was caused by the necessity to open registry cases before the actual date of hospital admission. This was to ensure that all patients had the opportunity to complete the questionnaires during the short two-day-period of hospitalisation. However, this procedure also leads to the fact that many cases have to be closed again due to a no-show or refusal to consent or that surveys were incomplete or were filled out inconsistently for linguistic reasons.

## Conclusion

The results of this study underscore the importance of factors of social inclusion and participation for interprofessional rehabilitation programmes to manage chronic pain – especially when focussing on productivity loss. Therefore, we recommend that in interprofessional rehabilitation programmes, productivity should be understood from a systemic approach, including structural (system-related) factors. Future research should focus on how factors of social inclusion and participation can best be assessed, and effectively addressed in health care programmes. Furthermore, it seems important to us to qualitatively explore the complex mechanisms between pain, productivity and factors of social inclusion and participation in more detail.

## Data Availability

The datasets generated and analysed during the current study are not publicly available due to legal reasons but are available from the corresponding author on reasonable request.
